# Down-Regulation of Rad51 Activity during Meiosis in Yeast Prevents Competition with Dmc1 for Repair of Double-Strand Breaks

**DOI:** 10.1371/journal.pgen.1004005

**Published:** 2014-01-23

**Authors:** Yan Liu, William A. Gaines, Tracy Callender, Valeria Busygina, Ashwini Oke, Patrick Sung, Jennifer C. Fung, Nancy M. Hollingsworth

**Affiliations:** 1 Department of Biochemistry and Cell Biology, Stony Brook University, Stony Brook, New York, United States of America; 2 Department of Molecular Biophysics and Biochemistry, Yale University School of Medicine, New Haven, Connecticut, United States of America; 3 Department of Obstetrics, Gynecology and Reproductive Sciences, University of California, San Francisco, San Francisco, California, United States of America; National Cancer Institute, United States of America

## Abstract

Interhomolog recombination plays a critical role in promoting proper meiotic chromosome segregation but a mechanistic understanding of this process is far from complete. In vegetative cells, Rad51 is a highly conserved recombinase that exhibits a preference for repairing double strand breaks (DSBs) using sister chromatids, in contrast to the conserved, meiosis-specific recombinase, Dmc1, which preferentially repairs programmed DSBs using homologs. Despite the different preferences for repair templates, both Rad51 and Dmc1 are required for interhomolog recombination during meiosis. This paradox has recently been explained by the finding that Rad51 protein, but not its strand exchange activity, promotes Dmc1 function in budding yeast. Rad51 activity is inhibited in *dmc1*Δ mutants, where the failure to repair meiotic DSBs triggers the meiotic recombination checkpoint, resulting in prophase arrest. The question remains whether inhibition of Rad51 activity is important during wild-type meiosis, or whether inactivation of Rad51 occurs only as a result of the absence of *DMC1* or checkpoint activation. This work shows that strains in which mechanisms that down-regulate Rad51 activity are removed exhibit reduced numbers of interhomolog crossovers and noncrossovers. A hypomorphic mutant, *dmc1-T159A*, makes less stable presynaptic filaments but is still able to mediate strand exchange and interact with accessory factors. Combining *dmc1-T159A* with up-regulated Rad51 activity reduces interhomolog recombination and spore viability, while increasing intersister joint molecule formation. These results support the idea that down-regulation of Rad51 activity is important during meiosis to prevent Rad51 from competing with Dmc1 for repair of meiotic DSBs.

## Author Summary

Sexual reproduction involves the generation of chromosomally balanced gametes through the specialized cell division of meiosis. A critical component of meiosis is the physical connection of homologous chromosomes through a combination of recombination and sister chromatid cohesion that is necessary for proper chromosome segregation at the first meiotic division. Meiotic recombination is initiated by the introduction of programmed double strand breaks (DSBs) that are processed and bound by RecA-like proteins called recombinases. In vegetative cells, the Rad51 recombinase preferentially mediates strand invasion of sister chromatids, while in meiotic cells, the meiosis-specific Dmc1 recombinase preferentially invades homologs. How Rad51 and Dmc1 activities are coordinated to generate interhomolog recombinants is a key question in meiosis. This work demonstrates that down-regulation of Rad51 activity is important when interhomolog recombination is occurring to prevent Rad51 from competing with Dmc1 for repair of meiotic DSBs. Premature activation of Rad51 results in increased intersister recombination and chromosome missegregation, producing inviable gametes. The evolutionary conservation of both Rad51 and Dmc1 suggests that down-regulation of Rad51 during meiosis may be important in metazoans as well as yeast.

## Introduction

Meiotic recombination is a highly conserved process that is critical for the accurate segregation of homologous chromosomes to opposite poles at the first meiotic division. Crossovers between non-sister chromatids, in combination with sister chromatid cohesion, physically connect homologous chromosomes, thereby allowing them to align properly at Metaphase I [1]. In the absence of crossovers, homologs segregate randomly to generate chromosomally imbalanced gametes, resulting in infertility or birth defects [2].

Meiotic recombination is initiated by the introduction of programmed double-strand breaks (DSBs) catalyzed by a meiosis-specific, evolutionarily conserved, topoisomerase-like protein, Spo11 [3]. Resection of the 5′ ends of the breaks results in 3′ single stranded tails. In many organisms, including yeast and mammals, these single stranded ends are bound by two conserved RecA-like recombinases, Rad51 and Dmc1, to form nucleoprotein filaments that mediate strand invasion of homologous DNA duplexes [4], [5]. Rad51 is present in both vegetative and meiotic cells, while Dmc1 is meiosis-specific. Subsequent processing of the resulting recombination intermediates results in the formation of either crossover or non-crossover chromosomes [6].

Rad54 and Rdh54/Tid1 are key accessory factors that physically interact with Rad51 and Dmc1 [7]–[10]. These paralogs are members of the Swi/Snf chromatin remodeling family of proteins and affect multiple steps of recombination, including stabilization of the Rad51 filament, the promotion of strand invasion, and the removal of recombinases after strand invasion to allow extension of the 3′ ends by DNA synthesis [11]. Rdh54 is also important in meiosis for removing Dmc1 from double stranded DNA that has not experienced DSBs [12]. There can be crosstalk between Rad54/Rdh54 and Rad51/Dmc1 (meaning, for example, that Rad54 may function with both Rad51 and Dmc1), as *rad54*Δ *rdh54*Δ double mutants have more severe meiotic phenotypes than either *rad54*Δ or *rdh54*Δ alone [13], [14]. However, recent biochemical studies using recombinant Dmc1, Rad51, Rad54 and Rdh54 proteins have demonstrated that Dmc1-Rdh54 and Rad51–Rad54 work as functionally distinct pairs for strand invasion *in vitro* [15].

In each meiosis, there are ∼160 DSBs generated by Spo11 [16]. The majority of these breaks are repaired by interhomolog interactions either as crossovers (COs) or noncrossovers (NCOs), while the remainder are presumably repaired using sister chromatids as templates [17], [18]. In yeast, physical analyses of joint molecule recombination intermediates have indicated a bias of approximately 4∶1 for interhomolog versus intersister recombination events [19]. This bias for interhomolog recombination is unique to meiosis: in vegetative cells, Rad51 acts to repair DNA damage preferentially using sister chromatids [20], [21].

Axial elements are generated when sister chromatids condense through the formation of loops tethered at their bases by meiosis-specific proteins [22]. In yeast, these proteins include Hop1, Red1 and Mek1 [23]–[25]. Diploids containing deletions of these genes exhibit reduced interhomolog recombination and increased levels of intersister recombination, demonstrating the importance of axial element components in promoting interhomolog recombination [26]–[31]. While Hop1 and Red1 are structural chromosomal components of axial elements, Mek1 is a serine/threonine protein kinase [32].

Prior to DSB formation, sequences within the loops of DNA created during axial element formation are recruited to the axes to form “tethered loop axis complexes” [33], [34]. Breaks are then created on the axes where Red1, Hop1 and Mek1 are present. This is important because DSBs result in the localized activation of Mek1 kinase activity via checkpoint kinase Mec1 phosphorylation of Hop1 [32], [35], [36]. It has been proposed that one end of each break remains tethered to the sister chromatid while the other end forms a “tentacle” that searches for the homologous chromosome. Mek1 facilitates interhomolog interactions in part by antagonizing sister chromatid cohesion mediated by meiosis-specific cohesin complexes [27].

Although both *rad51*Δ and *dmc1*Δ reduce interhomolog recombination, additional phenotypes of *rad51*Δ and *dmc1*Δ mutants are different, suggesting they play discrete roles in meiotic recombination [19], [37]–[39], [40]. *RAD51* is required to efficiently recruit Dmc1 to DSBs [37], [41]. In budding yeast, strains in which Dmc1 is the only recombinase (i.e. *rad51*Δ mutants) do not show interhomolog bias, indicating that the presence of the Rad51 protein is necessary to promote Dmc1 strand invasion of homologs [19], [38], [42]. This idea was confirmed by studies using a mutant of *RAD51* that is specifically defective in strand exchange. This mutant, *rad51-II3A*, exhibits a wild-type ratio of interhomolog∶intersister joint molecules, indicating that the presence of inactive Rad51 protein is sufficient for Dmc1 to mediate the bulk of meiotic recombination [43].

DSBs in *dmc1*Δ mutants are not repaired and become hyperresected in the SK1 strain background where the phenotype is most severe [44]. These breaks trigger the meiotic recombination checkpoint and cells arrest prior to the Meiosis I division [45]. Rad51 foci persist in *dmc1*Δ diploids, indicating the recombinase is present at DSBs but is not able to repair them [37], [40]. Inactivation of an analog-sensitive version of Mek1, called Mek1-as, after DSBs have formed in *dmc1*Δ cells allows Rad51-dependent repair of the breaks using sister chromatids [32], [46]. Therefore Mek1 phosphorylation of protein substrates is essential to inhibit Rad51 activity in the absence of *DMC1*.

A key step in downregulating Rad51 activity in *dmc1*Δ diploids is the inhibition of Rad51–Rad54 complex formation. There are two independent, meiosis-specific mechanisms by which formation of this complex can be suppressed. First, a meiosis-specific protein called Hed1 binds to Rad51 and excludes Rad54 [47], [48]. Ectopic expression of *HED1* in vegetative cells makes cells sensitive to DNA-damaging agents, indicating that the presence of Hed1 is sufficient to impair DNA repair by Rad51 [48]. Second, Rad54 is an *in vivo* target of Mek1. Phosphorylation of threonine 132 on Rad54 reduces the affinity of Rad54 for Rad51 *in vitro*, as the negative charge conferred by phosphorylation makes Rad51–Rad54 complex formation more difficult [49]. Consistent with this idea, substitution of aspartic acid, a negatively charged, phosphomimetic amino acid, for Rad54 threonine 132 increases sensitivity to DNA damaging agents in vegetative cells, while substitution with either alanine or lysine does not. Conditions that allow formation Rad51–Rad54 complexes in meiosis, such as over-expression of *RAD51* or *RAD54*, deletion of *HED1* or mutation of *RAD54-T132* to alanine, result in partial suppression of the *dmc1*Δ interhomolog recombination and spore viability defects [48]–[51]. Rad51-mediated interhomolog recombination in these strains requires Mek1, indicating there are additional Mek1 substrates that direct filaments containing Rad51–Rad54 to homologs [49].

Much of the work looking at Rad51 regulation has occurred in the *dmc1*Δ strains, as it simplifies the analysis to have only one recombinase present. However, the fact that *hed1*Δ and/or *RAD54-T132A* have little to no effect on spore viability in the presence of wild-type *DMC1* raises the question of whether down-regulation of Rad51 activity is part of normal meiosis [48], [49]. Several proposals have been made that suggest inhibition of Rad51 activity is specific to *dmc1*Δ: (1) Hed1 only inactivates Rad51 when *DMC1* is absent, (2) Mek1 regulation of Rad54 occurs only under checkpoint induced conditions and (3) the accumulation of single stranded DNA from the failure of DSB repair in *dmc1*Δ mutants causes hyperactivation of Mek1, resulting in a global inhibition of recombination [27], [48], [52]. This work addresses the question of whether down-regulation of Rad51 is important in wild-type meiosis by examining recombination in situations where Rad51 is activated but where Dmc1 is still functional and meiotic checkpoint arrest does not occur. We demonstrate that under certain conditions, Rad51, if not down-regulated, is capable of competing with Dmc1 during meiosis for DSB repair in yeast. We propose that down-regulation of Rad51 activity is therefore important to promote Dmc1-dependent interhomolog recombination.

## Results

### Mutation of a Conserved Amino Acid Creates a Hypomorphic *dmc1* Mutant

The use of *dmc1*Δ to study Rad51 inhibition has raised the possibility that it is the absence of *DMC1* or a pathological situation created by checkpoint arrest that is responsible for the down-regulation of Rad51 activity. One way to circumvent this problem is to create a mutant of *DMC1* that is still able to function in interhomolog recombination and therefore does not arrest, but is less efficient than wild type. If Rad51 and Dmc1 can compete for repair of DSBs, then combining up-regulated Rad51 with a hypomorphic version of *DMC1* may result in reduced interhomolog recombination and spore viability.

A hypomorphic *DMC1* allele was generated by mutation of threonine 159 to alanine. This threonine is highly conserved in both the Dmc1 and Rad51 protein families [53] (A. Neiman, personal communication). T159 is located two amino acids away from a conserved glutamate that is required for hydrolysis of ATP. In addition, T159 is equivalent to T164 in human Dmc1, which is located immediately adjacent to an asparagine (N163) that is important for monomer-monomer contacts [53]. We therefore hypothesized that the T159A mutation might make oligomerization of Dmc1 less efficient. Dmc1 interacts with itself in the two-hybrid system [7]. The T159A mutation was introduced into *DMC1* fused to either the Gal4 DNA binding domain (GBD) or the Gal4 activation domain (GAD), the plasmids were transformed into a strain containing a *GAL1-lacZ* reporter construct and protein-protein interactions were assessed by β-galactosidase assays. Consistent with published results, interaction between the two wild-type alleles of *DMC1* resulted in >100 units of β-galactosidase activity, while the control containing *GBD-DMC1* and *GAD* alone, exhibited background levels of activity [7] (Table 1). Plasmid combinations containing *DMC1/dmc1-T159A* exhibited only ∼30% the levels of wild-type β-galacotosidase activity, while no interaction was detected in cells containing both *GBD-dmc1-T159A* and *GAD-dmc1-T159A*. These results support the idea that the substitution of threonine 159 for alanine affects Dmc1 oligomerization.

**Table 1:** Two-hybrid interactions between *DMC1* and *dmc1-T159A*.

Y190/plasmids	Relevant genotype	β-gal units^a^
pMDE422/pACTII	*GBD-DMC1/GAD*	0.02+/−0.01
pMDE422/pMDE467	*GBD-DMC1/GAD-DMC1*	104.00+/−23.00
pMDE422/pMDE467-T159A	*GBD-DMC1/GAD-dmc1-T159A*	28.00+/−9.00
pMDE422-T159A/pMDE467	*GBD-dmc1-T159A/GAD-DMC1*	31.00+/−6.00
pMDE422-T159A/pMDE467-T159A	*GBD-dmc1-T159A/GAD-dmc1-T159A*	0.03+/−0.01

^a^ Two duplicate samples were assayed for three independent transformants. The values for each transformant were then averaged and the standard deviation calculated. β-galactosidase activity is indicated in Miller units [89].

**Figure 1:**
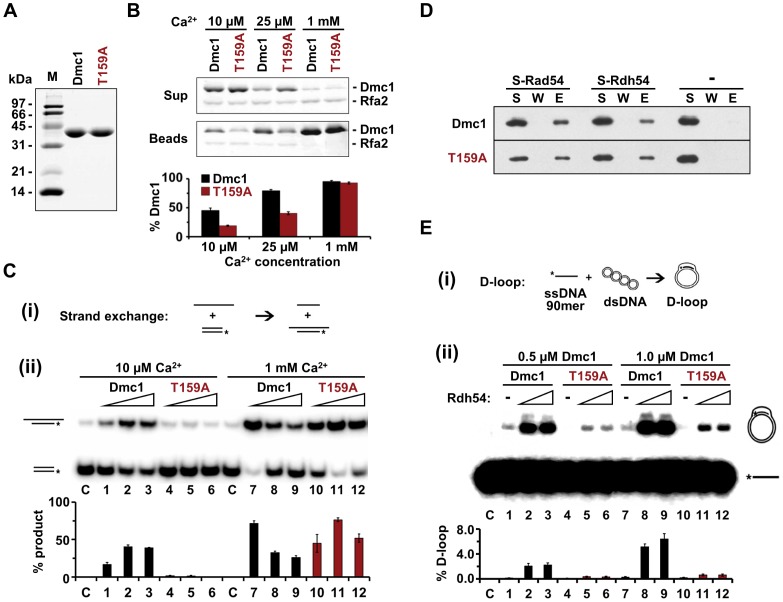
Biochemical characterization of recombinant Dmc1-T159A protein. A. For Dmc1 and Dmc1-T159A, 2.5 µg of each protein were analyzed by SDS-polyacrylimide gel electrophoresis (PAGE) and Coomassie staining. B. The stability of Dmc1 filaments on bead-immobilized ssDNA was assessed by exposing the filaments to RPA and measuring the amount of Dmc1 retained on the beads (n = 3, +/− standard error). Proteins were monitored by SDS-PAGE and Coomassie staining. The experiments were performed with different Ca^2+^ concentrations as indicated. Rfa2, the 30 kDa subunit of RPA is indicated. The histogram shows the percent of Dmc1 protein that remained associated with the beads after challenge by RPA as determined by band desitometry. C. (i) Schematic of the strand exchange recombination assay. (ii) Strand exchange activity of Dmc1 and Dmc1-T159A was monitored using 2, 4, or 8 µM protein in the presence of either 10 µM or 1 mM Ca^2+^. The histogram indicates the percent of radioactively labeled oligonucleotide that was incorporated into the slower migrating product by strand exchange (n = 3, +/− standard error). At 1 mM Ca^2+^, the inhibition of strand exchange seen at elevated concentrations of wild-type Dmc1 is likely due to coating of the dsDNA substrate by the recombinase, thereby blocking access of the ssDNA filament. D. Dmc1 and Dmc1-T159A interactions with Rad54 and Rdh54 were assayed by pull-down experiments. S-tagged Rad54 or Rdh54 (2 µg each) was incubated with 1.2 µg Dmc1 or Dmc1-T159A. The S-tagged protein was captured on S-protein agarose resin, which was washed and the protein eluted with SDS. S = supernatant after collecting the beads, W = supernatant obtained from washing the beads, E = eluate from beads. The “-” indicates that no tagged protein was added to the reaction. E. (i) Schematic of the D-loop recombination assay. (ii) Homologous DNA pairing activity of Dmc1 and Dmc1-T159A was assessed by a D-loop formation assay (n = 3, +/− standard error). 0.5 or 1.0 µM of Dmc1 or Dmc1-T159A was combined with 0, 150, or 250 nM of Rdh54. The histogram shows the percent of radioactive ssDNA that is incorporated into the slower migrating D-loop product via homologous pairing.

To determine whether any biochemical properties are affected by the T159A mutation, His_6_-tagged Dmc1 and Dmc1-T159A were purified from bacteria using a new procedure developed in the Sung laboratory [54]. Both wild-type and mutant proteins gave equivalent yields of good purity, indicating that the T159A mutation does not destabilize the protein (Figure 1A). To look at filament stability, an RPA competition assay was used [55]. This assay involves preloading Dmc1 onto single stranded (ss) DNA that is attached to magnetic beads through a biotin-streptavidin connection, adding RPA, a single-stranded DNA binding protein complex, and monitoring the amount of Dmc1 that is retained on the beads. If Dmc1 is stably bound to the ssDNA, then it will not be competed off by RPA. The experiment was performed under three different Ca^2+^ conditions. High Ca^2+^ (1 mM) has previously been shown to stabilize Dmc1 filaments [56]. The bulk of both the Dmc1 and Dmc1-T159A proteins remained associated with the ssDNA/beads under high Ca^2+^ conditions (Figure 1B). When the Ca^2+^ concentration was lowered to either 10 or 25 µM, RPA was able to compete off the Dmc1-T159A protein more readily than wild type, indicating that filaments made from the mutant protein are less stable (Figure 1B). To test whether this filament instability affects Dmc1-T159A activity, strand exchange reactions were performed. In the strand exchange reaction, one strand of a piece of duplex DNA is radioactively labeled and the duplex DNA is then incubated with an unlabeled single strand of DNA along with the recombinase (Figure 1C, panel i). Exchange of the unlabeled strand for the radioactive strand in the duplex is then detected by slower migration on polyacrylamide gels. Consistent with a lack of stable filament formation, only background levels of product were observed for Dmc1-T159A in strand exchange reactions at 10 µM Ca^2+^ (Figure 1C, panel ii). This defect is due to unstable filaments and not to a defect in catalytic function, as a wild-type level of strand exchange activity for Dmc1-T159A is restored at 1 mM Ca^2+^, where the filaments are stabilized.

Dmc1 exhibits physical interactions with Rad54 and Rdh54 both *in vitro* and *in vivo* [7], [15], [57]. Pull-down experiments show that Dmc1-T159A interacts as well as wild-type Dmc1 with both Rad54 and Rdh54 (Figure 1D). Homologous pairing activity can be assayed by examining D-loop formation, where a radioactively labeled single strand of DNA is annealed to a complementary sequence within a circular plasmid, thereby displacing the sequence of like polarity (Figure 1E, panel i). The ability of Dmc1 to form D-loops is stimulated by Rdh54 [15]. For Rad51, *RDH54* mutants specifically defective in interactions with the recombinase are unable to stimulate Rad51's homologous pairing activity [58]. Therefore, the observation that Rdh54 stimulates D-loop formation mediated by Dmc1-T159A indicates that Rdh54 is associated with the Dmc1-T159A presynaptic filament in a functionally competent manner (Figure 1E, panel ii). A reduced amount of D-loop product formed by Dmc1-T159A compared to Dmc1, both with and without Rdh54, is observed because D-loop formation is a more stringent measure of homologous pairing than the strand exchange assay in Figure 1C. We conclude that Dmc1-T159A is able to mediate homologous pairing but is less efficacious than wild type, presumably due to less efficient filament formation.

**Figure 2:**
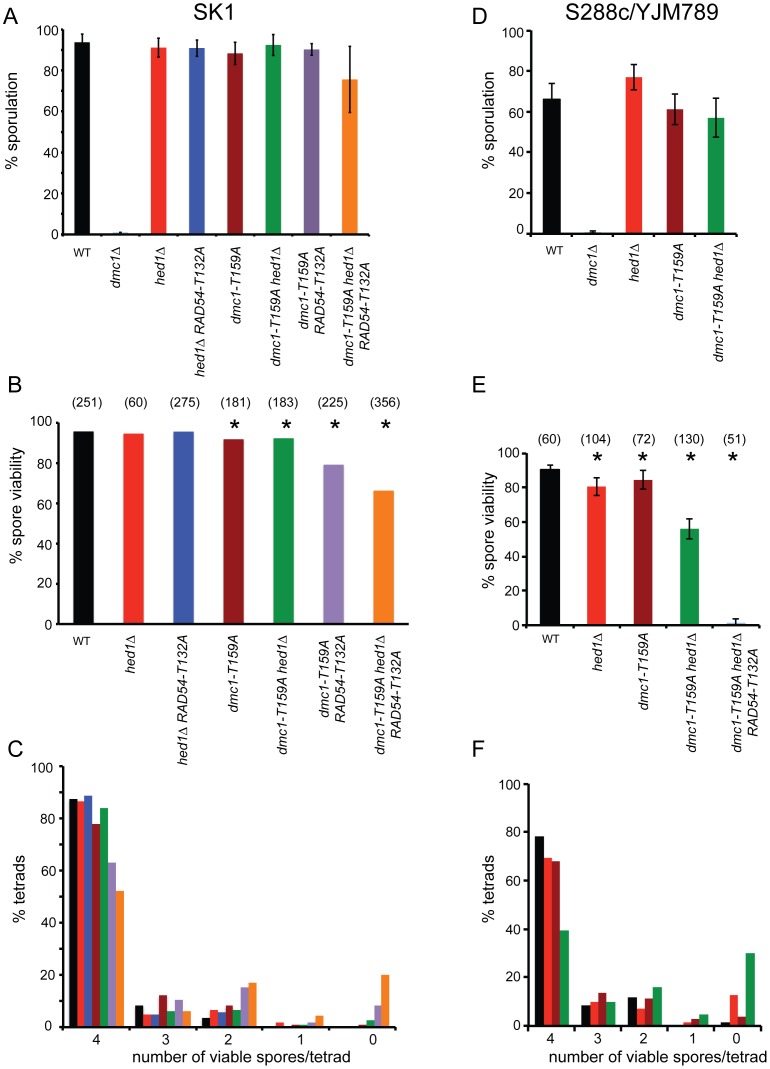
Characterization of various meiotic phenotypes in diploids containing *dmc1-T159A*. A. Sporulation in SK1 diploids: Wild-type (NH716), *dmc1*Δ (NH792), *hed1*Δ (NH1065), *hed1*Δ *RAD54-T132A* (NH1065::pHN104(S/N)^2^, *dmc1-T159A* (NH792::pNH301-T159A^2^), *dmc1-T159A hed1*Δ (NH942::pNH301-T159A^2^), *dmc1-T159A RAD54-T132A* (NH2231) and *dmc1-T159A hed1*Δ *RAD54-T132A* (NH2184) cells were transferred to Spo medium on plates for two days at 30°C and the percent sporulation was determined by phase contrast microscopy. 200 cells from at least four independent colonies were examined. Error bars represent the standard error. B. Spore viability in SK1 strains was assayed by tetrad dissection from at least four independent colonies. Numbers in parentheses indicate the number of tetrads dissected. * indicates that the spore viability is statistically significantly different from wild type by χ^2^ analysis. The *p* values are *dmc1-T159A* (<0.001); *dmc1-T159A hed1*Δ (<0.006); *dmc1-T159A RAD54-T132A* (<0.0001); *dmc1-T159A hed1*Δ *RAD54-T132A* (<0.0001) C. The distribution of viable spores in tetrads from the asci dissected for Panel B. D. Sporulation in S288c/YJM789 diploids: Wild-type (NH1053), *dmc1*Δ (NH2030), *hed1*Δ (NH2038), *dmc1-T159A* (NH2142), *dmc1-T159A hed1*Δ (NH2145), and *dmc1-T159A hed1*Δ *RAD54-T132A* (NH2146) cells were assayed for sporulation after four days on Spo plates at 30°C. E. Spore viability was assayed by dissection of at least 3 independent colonies. * indicates that the spore viability is statistically significantly different from wild type by χ^2^ analysis. The *p* values are *hed1*Δ (<0.001); *dmc1-T159A* (<0.02); *dmc1-T159A hed1*Δ (<0.0001); *dmc1-T159A hed1*Δ *RAD54-T132A* (<0.0001). F. The distribution of viable spores in tetrads from the asci dissected for Panel E.

**Figure 3:**
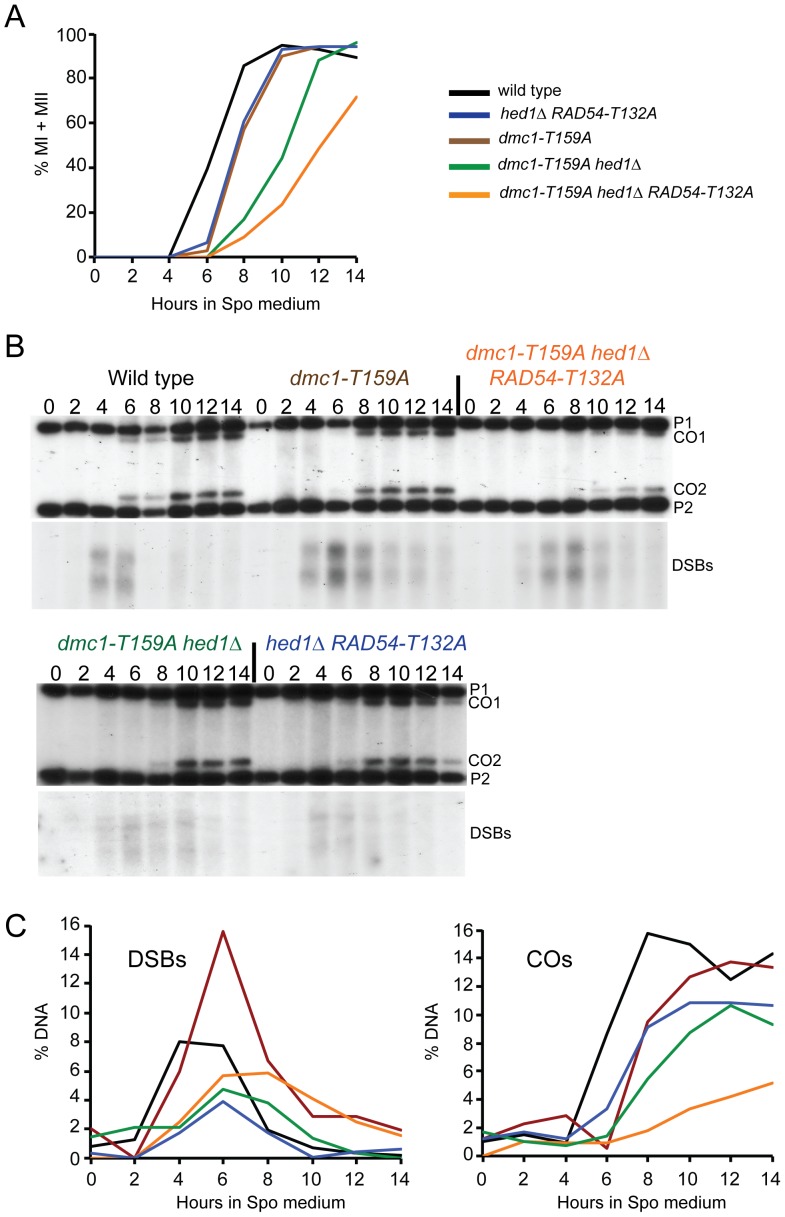
Meiotic progression and crossover formation in various *dmc1-T159A* SK1 strains. Wild-type, *hed1Δ RAD54-T132A*, *dmc1-T159A*, *dmc1-T159A hed1*Δ and *dmc1-T159A hed1*Δ *RAD54-T132A* diploids were transferred to Spo medium at 30°C at 0 hr and samples were taken at two hour intervals. Color coding is the same as in Figure 2. A. Meiotic progression was measured by staining the nuclei with DAPI and counting the fraction of bi-nucleate (MI) and tetranucleate (MII) cells. B. Crossovers and DSBs at the *HIS4/LEU2* hotspot. The DNA was digested with XhoI and probed as described in [64]. P1 and P2 represent the parental fragments and CO1 and CO2 represent the two products of reciprocal recombination. Numbers above each lane indicate the hours after transfer to Spo medium. C. Quantitation of the crossover and DSBs bands shown in Panel B. A replicate of this experiment is shown in Figure S1.

To examine whether the less stable filaments observed for Dmc1-T159A *in vitro* result in phenotypes *in vivo*, strains homozygous for *dmc1-T159A* were compared to wild type in two different strain backgrounds (SK1 and S288c/YJM789). *dmc1-T159A* rescues the sporulation defect of *dmc1*Δ in both backgrounds (Figure 2A and D). Furthermore, the spores produced by *dmc1-T159A* diploids have near wild-type levels of viability (Figure 2B and E). Meiotic progression is delayed ∼ two hours in the *dmc1-T159A* mutant (Figure 3A and S1A). DSBs arise with similar timing as wild type but accumulate to higher levels and persist longer, consistent with the idea that Dmc1-T159A is less efficient at DNA repair compared to Dmc1 (Figure 3B and C).

To test whether *dmc1-T159A* is defective in recombination, interhomolog crossovers were monitored by physical analyses using the *HIS4/LEU2* hotspot. The *HIS4/LEU2* hotspot is comprised of a DSB site flanked by restriction site polymorphisms that allow the detection of recombinant products using Southern blots [59]. In the wild-type diploid, crossovers were first detected at 6 hours (Figure 3B and C). Consistent with the meiotic progression data, crossovers in the *dmc1-T159A* diploid were reproducibly delayed by about two hours. Crossover levels are similar or only slightly reduced compared to wild type (Figure 3B and C; S1B and C).

The analysis of a single hotspot suggests that the Dmc1-T159A recombinase, although less efficient, is still able to produce nearly as many crossovers as wild-type. This idea was confirmed using a more global approach, whole genome sequencing, to examine all of the recombination events in individual tetrads. The *dmc1-T159A* mutation was introduced into two haploid strains (S288c and YJM789) that differ by 0.6% in their nucleotide sequences [17], [18]. All four spores from six *dmc1-T159A* tetrads were sequenced and then a software program called ReCombine was used to determine the numbers of COs and NCOs throughout the genome [60]. Because only four-spore viable tetrads were used for sequencing, a slight selection bias for meioses with higher numbers of COs theoretically may exist. However an actual bias has only previously been demonstrated when the sporulation frequency was particularly poor (<0.4% 4-spore asci) ([17], see *zip1* mutant), which is not the case here. (All sequences from this paper can be accessed at http://www.ncbi.nlm.nih.gov/bioproject/?term=PRJNA217886). In addition, the ReCombine data files are available from the Dryad Digital Repository: http://doi.org/10.5061/dryad.8gh60). The *dmc1-T159A* diploid exhibits 93% and 77% the wild-type levels of COs and NCOs, respectively (Table 2). The decrease in COs is not statistically significant, while the decrease in NCOs is. Crossover homeostasis occurs when the CO number is maintained at the expense of NCOs [61]. While the specific decrease in NCOs is suggestive that crossover homeostasis is occurring, one needs to look at the coefficient of variation (CV) and the correlation coefficient (CC) to determine whether CO homeostasis is intact. We are precluded from getting an accurate assessment of CV and CC because the sample size is too small.

**Table 2:** Various phenotypes obtained from sequencing tetrads from various strains derived from the S288c/YJM789 background.

Strain name	NH1053	NH2038	NH2142	NH2145
Relevant genotype	Wild type	*dmc1-T159A*	*hed1*Δ	*dmc1-T159A hed1*Δ
# tetrads sequenced	6	6	6	8
Average # COs/Tetrad	106.5+/−8.5^a^	90.8+/−12.6 *p* = 0.122	75.8+/−13.4 *p* = 0.001^b^	67.4+/−11.5 *p*<0.0001
Average # NCOs/tetrad	41.3+/−7.8^a^	25.3+/−6.1 *p* = 0.001	22.5+/−6.3 *p*<0.0001^b^	14.6+/−5.3 *p*<0.0001
Average # total IH events/tetrad	147.8+/−10.1	116.2+/−16.1	98.3+/−17.3	82.0+/−15.4
# E0 chr.	0	2	2	8
CO/NCO ratio	2.6	3.6 *p* = 0.051	3.4 *p* = 0.029^c^	4.6 *p*<0.0001
γ value^d^	2	2 *p* = 1.0	2.1 *p* = 1.0^e^	1.9 *p* = 0.9

^a^ Values indicate the standard deviation.

^b^ multicomparison Tukey test showing P values relative to wild type.

^c^ Z test of proportions.

^d^ The γ value is a measure of chromosome interference [17].

^e^ Bootstrapping analysis, applied as described in Ref. [93].

Crossovers are distributed throughout the genome by a process called interference, in which a crossover in one region lowers the probability of a crossover in an adjacent region [62]. Genome-wide data can be used to calculate interference by measuring inter-CO distances to calculate a value called γ. A lack of interference results in a value of γ = 1, while γ values >1 indicate positive interference [17]. The γ values for the wild-type and *dmc1-T159A* diploids are basically identical, indicating that the crossovers generated using the hypomorphic recombinase are distributed properly (Table 2). The *dmc1-T159A* mutant therefore provides a good tool for testing whether up-regulated Rad51 can compete with weakened Dmc1 *in vivo*.

### Elimination of Meiosis-Specific Constraints on Rad51–Rad54 Complex Formation Decreases Interhomolog Recombination in the Presence of *dmc1-T159A*

Combining *hed1*Δ and *RAD54-T132A* together, thereby removing the meiosis-specific constraints on Rad51–Rad54 interaction, has no deleterious effect on spore viability, suggesting that COs are not affected or only modestly affected (Figure 2B) [49]. Time course analysis confirms this prediction, with COs formed at the *HIS4/LEU2* hotspot at levels only 30% reduced from wild type (Figure 3B and C). There is, however, a delay in meiotic progression and CO formation in *hed1*Δ *RAD54-T132A* diploids, suggesting that allowing Rad51 and Rad54 to interact does affect meiotic recombination even in the presence of *DMC1* (Figure 3A). One explanation for the failure to see a strong CO defect in the *hed1*Δ *RAD54-T132A* diploid is that Dmc1 is a more efficient recombinase than Rad51 for interhomolog recombination. If true, then *hed1*Δ and/or *RAD54-T132A* may exhibit mutant phenotypes when combined with the less efficient *dmc1-T159A*. In SK1 strains, spore viability is similar for the *dmc1-T159A* and *dmc1-T159A hed1*Δ diploids, while a stronger decrease in spore viability was observed for *dmc1-T159A RAD54-T132A* (Figure 2B). Deletion of *HED1* in the *dmc1-T159A* diploid results in a modest decrease in COs, similar to what has previously been observed for *hed1*Δ alone [48] (Figure 3B and C). *hed1*Δ removes only one impediment to Rad51–Rad54 interaction, as Rad54 T132 phosphorylation by Mek1 is still occurring. Combining both *hed1*Δ and *RAD54-T132A* with *dmc1-T159A* results in a further decrease in spore viability (Figure 2B). The difference in spore viability between the triple mutant and either *dmc1-T159A hed1*Δ or *dmc1-T159A RAD54-T132A* is significant (χ^2^ analysis; *p*<0.0001), confirming the proposed functional redundancy between *HED1* and Rad54 T132 phosphorylation in the downregulation of Rad51 activity. Furthermore, the number of tetrads with four viable spores is decreased in the triple mutant with a corresponding increase in tetrads with either two or zero viable spores, a pattern indicative of Meiosis I chromosome non-disjunction (Figure 2C) [63]. This result suggests that up-regulating Rad51 activity does not interfere with repair (which would lead to broken chromosomes and a different spore viability pattern), but instead alters repair in such a way that interhomolog crossovers are decreased. In fact, COs are substantially reduced in the triple mutant compared to the other three strains (Figure 3B and C; S1B and C). Although the *dmc1-T159A hed1*Δ *RAD54-T132A* diploid sporulates well, addition of *RAD54-T132A* results in a further delay in meiotic progression than is observed for either *dmc1-T159A* or *dmc1-T159A hed1*Δ (Figure 3A and S1A).

In contrast to the SK1 background, the *dmc1-T159A hed1*Δ combination in the S288c/YJM789 background exhibits a synergistic decrease in spore viability compared to either *dmc1-T159A* or *hed1*Δ alone (Figure 2E). As with the SK1 diploid, the distribution of viable spores in the *dmc1-T159A hed1*Δ tetrads indicates that spore lethality is likely due to Meiosis I nondisjunction (Figure 2F). *dmc1-T159A hed1*Δ *RAD54-T132A* in the sequencing background reduced spore viability even further, making it difficult to obtain four viable spores for sequencing (Figure 2E). Therefore, we were unable to analyze the triple mutant using the sequencing assay. Sequence analysis of the genomes from the spores of eight *dmc1-T159A hed1*Δ tetrads shows statistically significant reductions in both COs and NCOs to 68% and 48% of the wild-type levels, respectively (Table 2). The γ value for *dmc1-T159A hed1*Δ is identical to wild type, however, indicating that interference is normal. Chromosomes without any crossovers are defined as E0 chromosomes. In 26 wild-type tetrads analyzed by Chen et al. (2008) and the six additional tetrads included here, no E0 chromosomes were observed. In contrast, eight E0 chromosomes were observed in the eight tetrads sequenced for *dmc1-T159A hed1*Δ (Table 2).

The delay in meiotic progression observed for *dmc1-T159A* indicates that the checkpoint may still be activated by the inefficient recombinase, even though a pathological arrest is not occurring. To test whether *hed1*Δ alone affects recombination, the genomes from six *hed1*Δ tetrads were sequenced. In this strain, *DMC1* is wild-type so the meiotic recombination checkpoint should not be triggered.

A small, but statistically significant reduction in spore viability was observed in the *hed1*Δ sequencing diploid, indicating this genetic background is more sensitive to the absence of *HED1* than SK1 or BR strains (Figure 2E). The average numbers of COs and NCOs were reduced, exhibiting 78% and 57% of the wild-type levels, respectively (Table 2). These reductions are statistically significant. This reduction is likely due to a change in repair and not because fewer breaks are being initiated, as *hed1*Δ does not affect DSB formation [48]. Interference is functioning, as indicated by the γ value. Two E0 chromosomes were observed for the six *hed1*Δ tetrads. This number is less than the 8 E0 chromosomes exhibited by *dmc1-T159A hed1*Δ. While this difference is intriguing, the sample size is currently too small to definitively say whether this difference is meaningful or not.

### Up-Regulation of Rad51 Activity in the *dmc1-T159A* Background Increases Intersister Recombination

**Figure 4:**
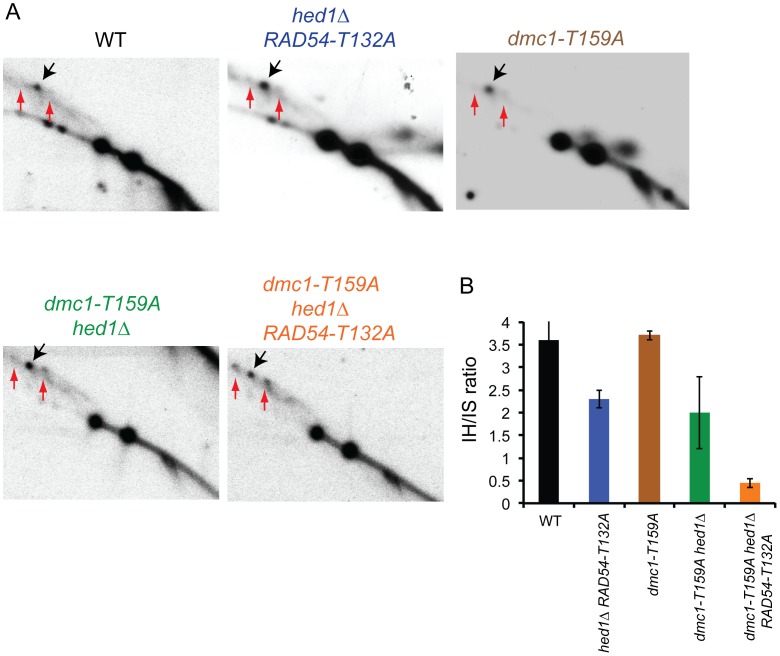
Meiotic joint molecule analysis in various SK1 *dmc1-T159A ndt80*Δ strains. *ndt80*Δ (NH2188), *hed1*Δ *ndt80*Δ *RAD54-T132A* (NH2223::pHN104(S/N)^2^, *dmc1-T159A ndt80*Δ (NH2235), *dmc1-T159A hed1*Δ *ndt80*Δ (NH2190) and *dmc1-T159A hed1*Δ *ndt80*Δ *RAD54-T132A* (NH2193) diploids were transferred to Spo medium for nine hours to arrest the cells in pachytene and the DNA was crosslinked with psoralen, extracted and digested with XhoI. Color coding is the same as in Figure 2. A. Southern blots of two-dimensional gels probed to detect interhomolog JMs (indicated by black arrows) and intersister JMs (indicated by red arrows) as described in [64]. B. Quantitation of the ratio of interhomolog:intersister joint molecules in the gels shown in A averaged with a second replicate. Error bars indicate the standard error.

The *dmc1-T159A hed1*Δ *RAD54-T132A* SK1 diploid exhibits decreased COs and spore viability with increased Meiosis I nondisjunction. One explanation for these results is that allowing Rad51–Rad54 complex formation in the presence of a less efficient Dmc1 results in more intersister repair. This idea was directly tested by examining formation of interhomolog and intersister joint molecules by two-dimensional gel analysis using the *HIS4/LEU2* hotspot. To prevent joint molecules from coming apart by branch migration, the DNA was crosslinked with psoralen prior to extraction [64]. After digestion with XhoI, the DNA was separated in one dimension by mass and in the second dimension by mass and shape. Probing a Southern blot of the DNA then reveals three spots that represent one interhomolog joint molecule (JM) intermediate flanked by the two intersister JM intermediates. Because DSB repair occurs at different rates in the different mutant diploids, this analysis was carried out in strains deleted for the middle meiotic gene transcription factor, *NDT80*. *ndt80*Δ diploids arrest in pachytene with unresolved double Holliday junctions, thereby allowing JMs to accumulate [65]–[68]. Cells were arrested after incubation in Spo medium for nine hours and the samples processed. In the *ndt80*Δ diploid, an ∼4∶1 ratio of interhomolog∶ intersister joint molecules was observed, consistent with the literature [19] (Figure 4). The IH∶IS ratio was reduced approximately two-fold by the combination of *hed1*Δ and *RAD54-T132A*. Therefore removing the meiosis-specific barriers to Rad51–Rad54 interaction does increase intersister recombination, although not to the extent that an effect on spore viability is observed. A wild-type ratio was observed for the *dmc1-T159A* strain, demonstrating that this mutant affects the rate of recombination without affecting interhomolog bias (Figure 4). The two-fold reduction in interhomolog bias in the *dmc1-T159A hed1*Δ diploid is similar to that observed for *hed1*Δ *RAD54-T132A* but is accompanied by a mild spore viability defect (Figure 2B; Figure 4). These data support the interpretation that the reduction of interhomolog events observed by genomic sequencing in the absence of *hed1*Δ is due to more DSBs being repaired using sister chromatids as templates. Finally the interhomolog∶intersister JM ratio was reduced even further (eight fold) in the *dmc1-T159A hed1*Δ *RAD54-T132A* diploid, consistent with the more severe phenotypes observed for this strain (Figure 4).

## Discussion

Given that Rad51 strand exchange activity is not required for interhomolog recombination, the question arises as to whether having Rad51 active when interhomolog recombination is occurring is deleterious to the cell due to Rad51's preference for repairing DSBs using sister chromatids [14], [20], [21], [43]. In yeast, Rad51 is prevented from repairing DSBs in *dmc1*Δ mutants both by Hed1 and Mek1 phosphorylation of Rad54 [46], [69] [47]–[49]. However the lack of obvious phenotypes observed for *hed1*Δ *RAD54-T132A* diploids has raised questions about whether down-regulation of Rad51 activity occurs normally during meiosis or whether it only occurs after triggering of the meiotic recombination checkpoint. One argument supporting the idea that Rad51 strand exchange activity is inhibited normally during meiosis is that phosphorylation of Rad54 T132 by Mek1 occurs during wild-type meiosis [70]. Furthermore cytological studies using wild-type cells show that Hed1 focus formation on chromosome is dependent upon *RAD51* (but not *DMC1*) and that 98% of Rad51 foci co-localize with Hed1 [48]. While these studies demonstrate that meiotic impediments to Rad51–Rad54 complex formation are present during wild-type meiosis, they do not address whether this downregulation is functionally important. To answer this question, whole genome sequencing of tetrads from a *hed1*Δ diploid was performed and revealed a decrease in both interhomolog COs and NCOs, even though *DMC1* is wild type. Furthermore, joint molecule experiments showed that intersister recombination is increased when down-regulation of Rad51 activity is abolished, particularly when combined with *dmc1-T159A*. These results indicate that regulation of Rad51–Rad54 complex formation by both Hed1 and Rad54 T132 phosphorylation normally occurs during meiosis.

In the sequencing strain background, *hed1*Δ decreases spore viability slightly on its own and synergistically in combination with *dmc1-T159A*, a phenotype that was not observed in SK1. The decreased spore viability of the double mutant is likely due, in part, to a greater reduction in crossovers compared to *hed1*Δ. In addition, the frequency of chromosomes without crossovers appears to be increased, although interference is wild-type. This suggests that, while the *dmc1-T159A hed1*Δ mutant is proficient in the distribution of crossovers along individual chromosomes, it may be defective in crossover assurance, which is the regulation that ensures that every chromosome sustains at least one crossover. If true, this could also account for the decreased spore viability relative to *hed1*Δ. Further work is necessary, however, to increase the sample size of E0 chromosomes before any definitive conclusions concerning this point can be made.

### Multiple Mechanisms Promote Interhomolog Bias during Meiosis

Crossovers between homologs are critical for proper alignment and segregation of homologous pairs of sister chromatids to opposite poles at the first meiotic division. This importance is underlined by the multiple, non-overlapping meiosis-specific mechanisms that cells use to change the bias for repair from sisters in vegetative cells to homologs in meiotic cells. First, in vegetative cells, intersister DSB repair is mediated by Rad51 and promoted by the recruitment of cohesin molecules to the DSBs [71]–[73]. In contrast, meiotic cells utilize cohesin complexes containing a meiosis-specific kleisin subunit, Rec8, and cohesin function at DSBs is antagonized by Mek1, thereby promoting interhomolog recombination [27], [74]. Second, the meiosis-specific axial element structures into which sister chromatids are packaged create additional constraints that promote interhomolog strand invasion in a variety of organisms. Functional orthologs of Hop1 in nematodes, plants and mammals all promote interhomolog bias, suggesting that at least some of the fundamental mechanisms for promoting crossovers between homologs during meiosis are conserved [75]–[79]. Third, many organisms utilize the meiosis-specific Dmc1 recombinase, which in budding yeast has been shown to be sufficient for the bulk of meiotic recombination [43], [80]. Dmc1 may be better at performing interhomolog recombination than Rad51 due to intrinsic properties of the protein. For example, the D-loops formed by Dmc1 are more resistant to dissociation than those formed by Rad51 [57]. In addition Dmc1 utilizes different accessory factors such as Rdh54 that promote interhomolog recombination [15]. This work demonstrates yet another mechanism for promoting interhomolog bias: the down-regulation of Rad51 activity by the prevention of Rad51–Rad54 interaction.

The Dmc1-T159A protein is defective in efficiently forming filaments while maintaining the ability to mediate strand exchange and interaction with Rad54 and Rdh54. The delay in DSB repair, crossover formation and meiotic progression confirms that this mutation makes Dmc1 less efficient as a recombinase. *dmc1-T159A* cells produce nearly wild-type levels of crossovers which are distributed normally throughout the genome, as well as being wild-type for interhomolog bias, consistent with biochemical experiments indicating that the mutant affects filament formation. One possibility is that it may take longer for Dmc1-T159A containing filaments to form, but having done so, they then function normally in recombination. It is important however, that although meiotic progression is delayed by *dmc1-T159A*, the cells do not arrest, thereby avoiding the proposed hyperactivation of Mek1 [52]. The fact that decreased spore viability, increased Meiosis I non-disjunction, and reduced interhomolog recombination are strongly apparent only when up-regulation of Rad51 activity was combined with the less efficient *dmc1-T159A* mutant, supports the idea that Dmc1 is fundamentally a more effective interhomolog recombinase than Rad51 in meiosis. The amino acid mutated in Dmc1, T159, is conserved in Dmc1 and Rad51 proteins from all species that have been analyzed, suggesting that it may be possible to construct similar hypomorphic mutants for these recombinases in other organisms.

Recent work has shown that DSBs occur on chromosome axes where localized activation of Mek1 is presumed to occur [34]. In plants the localization of Rad51 and Dmc1 to DSBs is asymmetric, with Rad51 loaded onto one end and Dmc1 loaded onto the other end [41]. The asymmetric loading model is appealing because of the asymmetry inherent in the recombination reaction that generates COs. During CO formation, the two ends of the DSB play different roles. The first end must undergo strand invasion of a homologous duplex, while the second end undergoes annealing to the newly displaced strand such that a double Holliday junction can subsequently be formed [6]. One model is that local antagonism of sister chromatid cohesion allows the release of one end of the DSB, presumably the one bound by Dmc1, while the other end remains tethered to the sister chromatid [27]. After the Dmc1 filament stably invades the homolog, the second end must be released to allow its annealing to the displaced strand at the nascent interhomolog joint. Whether this release results from a signal arising from individual recombination events, or is a globally regulated event, is not known.

We propose that preventing Rad51 from binding to Rad54 is important for keeping the second end quiescent until the first end has engaged the homolog. When *HED1* is removed and Rad54-T132 phosphorylation is prevented, Rad51 is now active. Although there is a two-fold increase in intersister recombination, the efficiency of Dmc1 in making stable connections with the homolog prevents a large reduction in crossovers. The inefficiency of Dmc1-T159A in mediating strand invasion (possibly due to a delay in filament formation) may provide the end containing Rad51 more time to mediate the stable strand invasion of the sister chromatid. This would then send the signal for the Dmc1-containing end to anneal to the displaced strand at the sister chromatid. The lack of coordination between the two ends could further slow DSB repair, explaining the meiotic delay of the *dmc1-T159A hed1*Δ *RAD54-T132A* diploid.

Although it has been suggested that Dmc1 and Rad51 are bound to different sides of DSBs in yeast, similar to the situation in plants [81], this has not been definitively established. In contradiction to this idea, recent experiments have shown that Rad51 can act as an accessory factor for Dmc1 *in vitro*, suggesting instead that the two recombinases may co-localize on the same ends of DSBs in yeast [43]. In this case, our model that activation of Rad51 allows one end to engage in intersister recombination still holds, although the mechanism that determines the asymmetry of the ends (strand invasion vs. annealing to the displaced strand) remains to be determined.

It should be noted that interhomolog recombination does not require that there be two recombinases. Organisms such as *Drosophila* and *C. elegans* do not contain *DMC1* in their genomes and therefore meiotic recombination is mediated solely by Rad51 [80]. Furthermore even in organisms that utilize both recombinases, mutants exist which allow interhomolog recombination to occur by either Rad51 alone or Dmc1 alone [41], [48], [49]. However, the frequencies of interhomolog recombination in these latter situations are not wild-type. Therefore, the optimal situation in organisms with both Rad51 and Dmc1 is to have both recombinases present. This may be because of a requirement for Rad51 to load Dmc1 or to stimulate Dmc1's enzymatic activities [41], [43], [81]. But while the presence of Rad51 is important for recombination, it is also important that Rad51's capacity to perform strand invasion be turned off while interhomolog recombination is occurring to prevent competition with Dmc1 for repair of DSBs.

## Materials and Methods

### Strains

The genotypes of all the strains used in this work can be found in Table 3. Genes were deleted by polymerase chain reaction (PCR)-based methods using the *kanMX6*, *natMX4* or *hphMX4* markers that confer resistance to G418, nourseothricin and Hygromycin B, respectively [82]–[84]. All deletions were confirmed by colony PCR. For genomic sequencing of tetrads, isogenic diploids were generated in which one parent is a derivative of S288c, JCF4413, and the other is a clinical isolate, YJM789 [17]. YJM789 has a tendency to become aneuploid, therefore manipulations of this strain were limited as much as possible. To create a diploid homozygous for *dmc1-T159A*, *DMC1* was first deleted from both haploids using *kanMX6*. The *dmc1-T159A URA3* plasmid, pNH301-T159A, was digested with HindIII to target integration upstream of the *DMC1* open reading frame, and transformed into JCF4413 dmc1. Transformants were grown in YPD and plated on 5-fluoro-orotic acid to select for popouts of the plasmid [85]. Strains that retained the *dmc1-T159A* allele were selected based on their sensitivity to G418. The presence of the *dmc1-T159A* allele was further confirmed by colony PCR. To introduce a second copy of *dmc1-T159A* into this haploid, pNH301-T159A was integrated as before upstream of *dmc1-T159A*. JCF4413 dmc1-T159A::URA3::dmc1-T159A was then mated to YJM789 dmc1 to make the diploid, NH2142. A similar procedure was followed using JCF4413 dmc1 hed1 and YJM789 dmc1 hed1 to make NH2145. Prior to sequencing, each spore colony was checked using allele-specific colony PCR to confirm that no aneuploidies had arisen during the growth of the strains [86].

**Table 3:** *S. cerevisiae* strains^a^.

Name	Genotype	Source
YJM789	*MATα ho::hisG lys2 cyh^R^*	[94]
JCF4413	*MAT* **a** *lys5 ho::hisG ura3*Δ*::natMX4*	This work
NH1053	*MATαho::hisGlys2cyh^R^LYS5URA3 MAT* **a** *ho::hisG LYS2 CYH lys5 ura3*Δ*::natMX4*	This work
NH2030	NH1053 only *dmc1*Δ*::kanMX6*	This work
NH2038	NH1053 only *hed1*Δ*:::hphMX4*	This work
NH2142	same as NH1053 only *dmc1-T159A::URA3::dmc1-T159A dmc1*Δ*::kanMX6*	This work
NH2145	NH1053 only *dmc1-T159A::URA3::dmc1-T159A* hed1Δ*::hphMX4* dmc1Δ::kanMX6 hed1Δ::hphMX4	This work
NH2146	NH1053 only *dmc1-T159A* hed1Δ*::hphMX4* dmc1Δ::kanMX6 hed1Δ::hphMX4 RAD54::URA3::RAD54-T132A RAD54	This work
NH716	*MATα leu2::hisG his4-X::LEU2(NgoMIV+ori)* hoΔ*::hisG* ura3(Δ*pst-sma)* MAT***a*** * leu2::hisG HIS4::LEU2(BamH+oriI) ho*Δ*::hisG ura3(*Δ*pst-sma)*	[42]
NH792	NH716 only *dmc1*Δ*::kanMX6*	This work
NH1065	NH716 only *hed1*Δ*::natMX4*	This work
NH1065:: pHN104(S/N)^2^	NH716 only *hed1*Δ*::natMX4 RAD54::URA3-RAD54-T132A*	This work
NH792:: pNH301-T159A^2^	NH716 only *dmc1*Δ*::kanMX6::URA3-dmc1-T159A*	This work
NH942:: pNH301-T159A^2^	NH716 only *dmc1*Δ*::kanMX6::URA3-dmc1-T159A hed1*Δ*::natMX4*	This work
NH2231	NH716 only *dmc1-T159A RAD54::URA3::RAD54-T132A*	This work
NH2184	NH716 *dmc1-T159A hed1*Δ*::natMX4 RAD54::URA3::RAD54-T132A*	This work
NH2188	NH716 only *ndt80*Δ*::natMX4*	This work
NH2223:: pHN104(S/N)^2^	NH716 only *hed1*Δ*::natMX4 ndt80*Δ*::natMX4 RAD54::URA3-RAD54-T132A*	This work
NH2235	NH716 only *dmc1-T159A ndt80*Δ*::hphMX4*	This work
NH2190	NH716 only *dmc1-T159A hed1*Δ*::natMX4 ndt80*Δ*::hphMX4*	This work
NH2193	NH716 only *dmc1-T159A hed1*Δ*::natMX4 ndt80*Δ*::hphMX4 RAD54::URA3::RAD54-T132A*	This work
Y190	*MAT* **a** *leu2-3,112 gal4 gal80 his3 trp1-901 ade2-101 ura3-52::pGAL-lacZ::URA3 lys2::pGAL-HIS3::LYS2 cyh^R^*	[7]

^a^ Superscript “2” indicates that the plasmid was integrated into both haploid parents and the diploid is therefore homozygous.

The two-hybrid reporter strain,Y190, was generously provided by Michael Dresser (Oklahoma Medical Research Foundation). All other strains were derived from NHY1210 and NHY1215, SK1 strains that contain the *HIS4/LEU2* hotspot (generously provided by Neil Hunter). The wild-type diploid created by mating NHY1210 and NHY1215 is called NH716 [42]. Strains containing *dmc1-T159A* were generated by first substituting *DMC1* with *kanMX6* and then transforming the strains with pNH301-T159A to integrate *dmc1-T159A* upstream of *dmc1*Δ*::kanMX6*. For some strains, the *dmc1*Δ*::kanMX6* allele was recombined out as described above so that *dmc1-T159A* haploids could subsequently be transformed with pHN104(Sph1/NruI), which contains the *RAD54-T132A* allele [49].

### Plasmids

The *GBD-DMC1* and *GAD-DMC1* plasmids (pMDE422 and pMDE467, respectively; provided by Michael Dresser) contain codons 3-334 of *DMC1* fused to either the Gal4 DNA binding or activation domains [7]. The T159A mutation changes codon 159 from ACT to GCT and was introduced into various plasmids using the QuikChange kit (Stratagene). For purification of Dmc1 and Dmc1-T159A from *E.coli*, the T159A mutation was introduced into the *DMC1* expression plasmid, pNRB150scDMC1 [87], to generate pNRB150scDMC1-T159A. To introduce the *dmc1-T159A* mutation into yeast cells, *DMC1* was first cloned into a *URA3* integrating plasmid by moving a 2.4 kb NotI/XhoI fragment from pRS316-DMC1 (generously provided by J. Engebrecht) into NotI/XhoI-digested pRS306 [88]. The resulting plasmid, pNH301, was used for site-directed mutagenesis to make pNH301-T159A. All mutated alleles were sequenced at the Stony Brook University DNA Sequencing Facility to confirm that no unexpected mutations were introduced during the mutagenesis. Digestion of pNH301-T159A with HindIII targets the plasmid to integrate approximately 600 bp upstream of the *DMC1* gene. To introduce *RAD54-T132A*, pHN104(Sph1/NruI), a *URA3 RAD54-T132A* integrating plasmid, was digested with BsiWI to target integration upstream of *RAD54* and transformed into the appropriate haploids.

### Whole Genome DNA Sequencing

DNA was prepared from each spore colony as described in [86] and sequenced at either the Vincent J. Coates Genomics or University of California San Francisco Sequencing Facilities using the Illumina HiSeq 2000 platform with 50 nt single end reads. Analysis of the sequences was performed using the ReCombine suite of programs to determine the number of crossovers and non-crossovers and the interference values [60]. The sequencing data can be found at NCBI SRA Bioproject, Accession number PRJNA217886 (http://www.ncbi.nlm.nih.gov/bioproject/?term=PRJNA217886). The ReCombine user manual and software package can be accessed at http://sourceforge.net/projects/recombine/. The ReCombine data files used to determine the number of COs and NCOs are available from the Dryad Digital Repository: http://doi.org/10.5061/dryad.8gh60).

### Two-Hybrid Assays

Y190 was co-transformed with the indicated plasmids and protein-protein interactions were monitored using liquid β-galactosidase assays. Transformants were inoculated into 5 ml SD-leu-trp and grown overnight at 30°C. Cells were diluted 1∶10 in SD-leu-trp and the OD_600_ was measured to determine cell density. Two 1.5 ml aliquots of culture from each transformant were pelleted in microfuge tubes and washed once in Z buffer (60 mM Na_2_HPO_4_, 40 mM NaH_2_PO_4_.H_2_0, 10 mM KCl, 1 mM MgSO_4_.7H_2_0, 4 mM 2-mercaptoethanol). The cells were then resuspended in 150 µl Z buffer, vortexed and lysed by the addition of 50 µl chloroform and 20 µl 0.1% SDS. After vortexing for 30 sec, the tubes were equilibrated at 30°C for five minutes. 700 µl of 1.2 mg/ml ortho-Nitrophenyl-β-galactoside (ONPG) made up in Z buffer was added to each tube which were then placed at 30°C. As soon as a yellow color appeared, the time was noted and the reactions were stopped by the addition of 500 µl 1M Na_2_CO_3_ and put on ice. Any reactions that had not turned yellow by two hours were stopped at that time. Cells were pelleted and the OD_420_ of the supernatants was determined. Miller units were calculated using the following formula: (1000×A_420_)/(A_600_×time (min)×vol (ml) [89]. Two replicates from each transformant were averaged and these numbers were then used to calculate the average and standard deviation from three transformants.

### Protein Purification

Recombinant budding yeast Dmc1 proteins (WT and T159A) were purified according to a new procedure developed by the Sung laboratory [54], which results in Dmc1 preparations that have a higher specific recombinase activity than the published protocol [87]. Two independent preparations of Dmc1-T159A were analyzed to ensure consistency of results. The proteins were concentrated in an Amicon Ultra micro-concentrator (Millipore), snap-frozen in liquid nitrogen, and stored at −80°C. RPA was purified as described in [90]. S-Rad54 and S-Rdh54 were purified as described in [10] and [58], respectively.

### Strand Exchange Assay

Oligonucleotide-based DNA pairing and strand exchange assay was conducted as described previously [91]. Briefly, Dmc1 (2, 4, and 8 µM) was incubated with 150-mer ssDNA oligonucleotide (6 µM nucleotides) in 10.5 µl of buffer A (50 mM Tris-HCl, pH 7.5, 1 mM DTT, 20 mM KCl, 2 mM ATP, 5 mM MgCl_2_) with the indicated amount of CaCl_2_ for 5 min at 37°C. 1 µl of 50 mM spermidine and 1 µl of ^32^P-labeled homologous 40-mer dsDNA (for 0.8 µM base pairs final concentration) were added to initiate the reaction. The reactions were incubated for 30 min at 37°C. The samples were deproteinized by the addition of 1% SDS and 1 mg/ml proteinase K, and subjected to electrophoresis in a 10% polyacrylamide gel run in TAE buffer (40 mM Tris acetate, pH 7.4, 0.5 mM EDTA). Products were quantitated using a phosphoimager.

### RPA Challenge Assay to Test for Dmc1 Filament Stability

This assay was modified from one developed by [55] for Rhp51. Dmc1 protein (2.4 µM) was added to biotinylated dT 83-mer ssDNA (4.3 µM nucleotides) coupled to magnetic streptavidin beads (Roche) in 10 µl of buffer B (35 mM Tris-HCl, pH 7.5, 1 mM DTT, 20 mM KCl, 2 mM ATP, 5 mM MgCl_2_, 100 µg/ml BSA). The reactions were incubated at 37°C for 5 min to permit filament formation. Unbound Dmc1 protein was removed by magnetic separation, and 10 µl of buffer B with 0.4 µM of RPA was added to the beads. The reactions were mixed and incubated at 30°C for 5 min followed by magnetic separation of the supernatant and bead fractions. Both fractions were analyzed by SDS-PAGE, Coomassie Blue staining, and band densitometry. The indicated amount of CaCl_2_ was included in both the binding and RPA challenge buffers.

### Affinity Pull-Down Assay

Dmc1 (1.2 µg) was incubated with or without S-tagged Rad54 or S-tagged Rdh54 (2 µg each) in 30 µl of buffer C (40 mM K_2_HPO_4_, pH 7.5, 0.5 mM EDTA, 10% glycerol, 150 mM KCl, 0.01% IGEPAL, 1 mM DTT) for 30 min at 4°C. The reactions were mixed with 10 µl of S-protein agarose beads (Novagen) and incubated for 30 min at 4°C with agitation. Beads were washed twice with 200 µl of buffer C and bound protein was eluted with 2% SDS. Supernatant (S), elution (E), and wash (W) fractions were analyzed by 10% SDS-PAGE followed by western blot with α-hDMC1 antibody (Santa Cruz Biotechnology, catalog # 22768). This experiment was performed twice with similar results.

### D-loop Assay

The D-loop reaction was conducted as described [10], [87]. Briefly, Dmc1 (0.5 or 1.0 µM) was incubated with ^32^P-labeled 90-mer oligonucleotide substrate (1.5 µM nucleotides) at 37°C for 5 min. Next, Rdh54 (0, 150, or 250 nM) was added along with pBluescript SK replicative form I DNA (72 µM base pairs). The reaction (12.5 µl final volume) had a buffer composition of 50 mM Tris-HCl, pH 7.5, 1 mM DTT, 72 mM KCl, 1 mM MgCl_2_, 5 mM CaCl_2_, and 4 mM ATP with an ATP regenerating system (20 mM creatine phosphate, 30 µg/ml creatine kinase). After a 15 min incubation at 30°C, SDS (1%) and proteinase K (1 mg/ml) were added, followed by a 5 min incubation at 37°C. The deproteinized samples were subjected to electrophoresis in a 1% agarose gel and analyzed by phosphorimaging.

### Timecourses and Physical Analyses of Recombination

Cells were pregrown in YPA and transferred to Spo medium (2% potassium acetate) and incubated at 30°C as described in [92]. At the appropriate time points, an aliquot of cells was fixed with formaldehyde and stained with 4′,6-diamidino-2-phenylindole (DAPI). Meiotic progression was monitored by fluorescent microscopy to determine the number of binucleate (MI) and tetranucleate cells (MII). At least 200 cells were counted for each colony. Physical analyses were performed as described in [64]. For the one-dimensional gel analysis shown in Figure 3B, DNA was isolated using the MasterPure Yeast DNA Purification kit (Epicentre, Cat. # MPY80200). For the CO analysis shown in Figure S1 as well as the two-dimensional gel experiments, cells were treated with psoralen and crosslinked with ultra-violet light. DNA was extracted and digested with XhoI and probed after fractionation on a one-dimensional gel to look at crossover formation. To look at joint molecules, *ndt80*Δ diploids were arrested nine hours after transfer to Spo medium and the psoralen crosslinked, XhoI-digested DNA was fractionated in two-dimensions prior to probing on Southern blots. The interhomolog JMs were normalized to the total DNA, as was the sum of the two intersister JMs, and these values were used to calculate the interhomolog: intersister JM ratios. Quantitation was performed using the MultiGauge software with a Fujifilm FLA-7000 phosphoimager. Timecourses analyzing COs were conducted three times for all strains except NH1065 (*hed1*Δ *RAD54-T132A*), which was only examined twice. All five strains shown in Figure 3 are from the same timecourse, while for Figure S1, the NH1065 strain was performed on a different day as the other four strains. DSBs were only examined once in the Figure 3 timecourse.

## Supporting Information

Figure S1 Meiotic progression and crossover formation in various *dmc1-T159A* SK1 strains. Wild-type, *hed1*Δ *RAD54-T132A*, *dmc1-T159A*, *dmc1-T159A hed1*Δ and *dmc1-T159A hed1*Δ *RAD54-T132A* diploids were transferred to Spo medium at 30°C at 0 hr and samples were taken at two hour intervals. Color coding is the same as in Figure 2. A. Meiotic progression was measured by staining the nuclei with DAPI and counting the fraction of bi-nucleate (MI) and tetranucleate (MII) cells. B. Crossovers at the *HIS4/LEU2* hotspot. The DNA was digested with XhoI and probed as described in [64]. P1 and P2 represent the parental fragments and CO1 and CO2 represent the two products of reciprocal recombination. Numbers above each lane indicated the hours after transfer to Spo medium. C. Quantitation of the crossovers shown in Panel B.
(TIF)
