# Supplementary figures and images for: Down-Regulation of Rad51 Activity during Meiosis in Yeast Prevents Competition with Dmc1 for Repair of Double-Strand Breaks
Source: PLoS Genet. 2014 Jan 23;10(1):e1004005. doi: 10.1371/journal.pgen.1004005 (PMC3900393; doi:10.1371/journal.pgen.1004005)

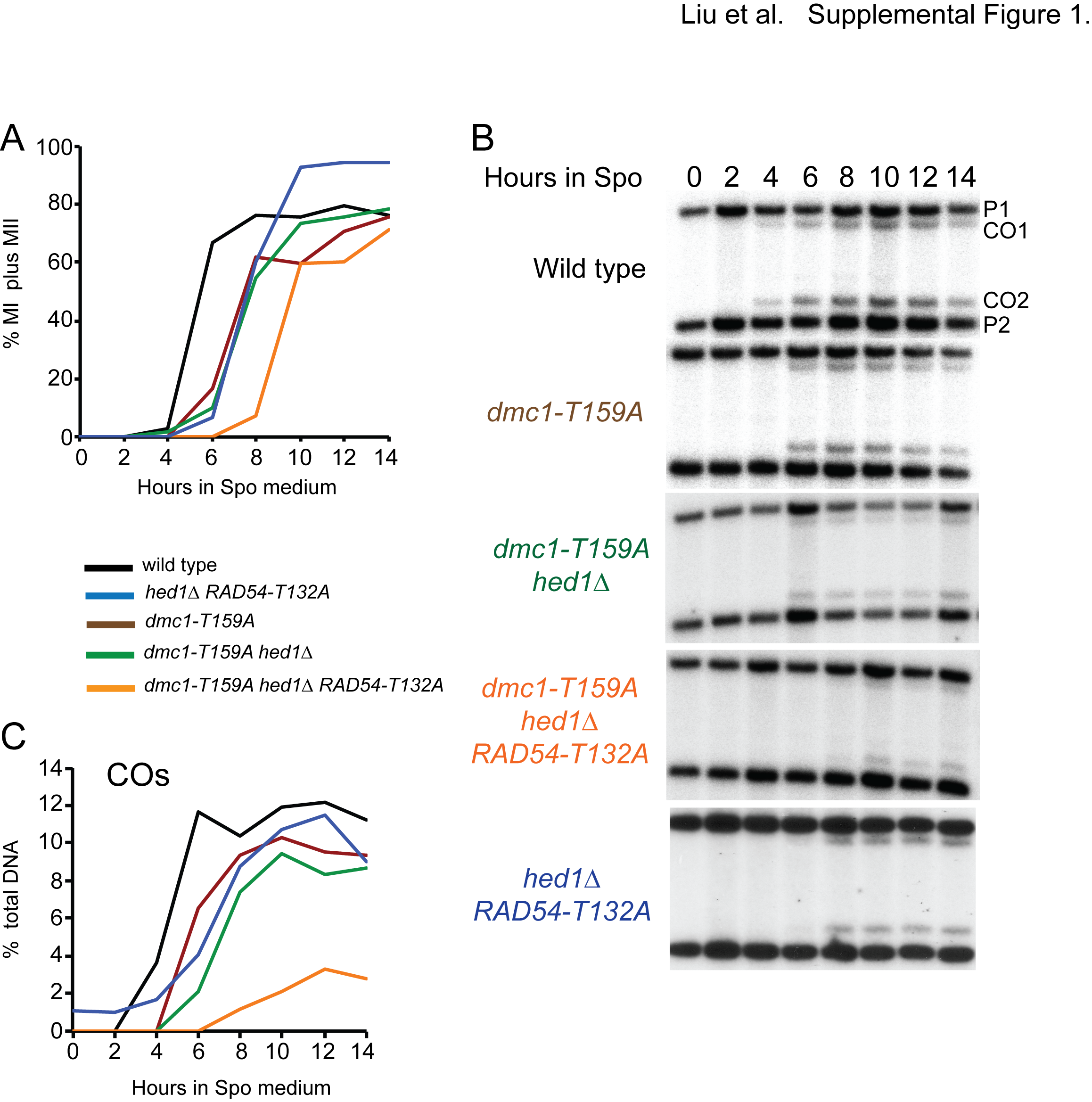

Supplement: Figure S1 — Meiotic progression and crossover formation in various dmc1-T159A SK1 strains. Wild-type, hed1Δ RAD54-T132A, dmc1-T159A, dmc1-T159A hed1Δ and dmc1-T159A hed1Δ RAD54-T132A diploids were transferred to Spo medium at 30°C at 0 hr and samples were taken at two hour intervals. Color coding is the same as in Figure 2. A. Meiotic progression was measured by staining the nuclei with DAPI and counting the fraction of bi-nucleate (MI) and tetranucleate (MII) cells. B. Crossovers at the HIS4/LEU2 hotspot. The DNA was digested with XhoI and probed as described in [64]. P1 and P2 represent the parental fragments and CO1 and CO2 represent the two products of reciprocal recombination. Numbers above each lane indicated the hours after transfer to Spo medium. C. Quantitation of the crossovers shown in Panel B. (TIF) [file pgen.1004005.s001.tif]
